# A propensity score matching analysis of survival following segmentectomy or wedge resection in early-stage lung invasive adenocarcinoma or squamous cell carcinoma

**DOI:** 10.18632/oncotarget.7284

**Published:** 2016-02-09

**Authors:** Yang Zhang, Yihua Sun, Haiquan Chen

**Affiliations:** ^1^ Department of Thoracic Surgery, Fudan University Shanghai Cancer Center, Shanghai, China; ^2^ Department of Oncology, Shanghai Medical College, Fudan University, Shanghai, China; ^3^ Shanghai Chest Hospital, Shanghai Jiao Tong University, China; ^4^ Institutes of Biomedical Sciences, Fudan University, Shanghai, China

**Keywords:** non-small cell lung cancer, wedge resection, segmentectomy, survival outcomes

## Abstract

**Purpose:** To compare the survival outcomes following segmentectomy or wedge resection in early-stage lung cancer.

**Methods:** A total of 5880 patients with invasive lung adenocarcinoma or squamous cell carcinoma from the Surveillance, Epidemiology, and End Results (SEER) database were included in this study, of which 1156 received segmentectomy. Baseline characteristics were balanced using propensity score methods. Cox regression analysis was used to compare overall survival (OS) and lung cancer-specific survival (LCSS) following segmentectomy or wedge resection after matching patients based on propensity scores.

**Results:** Overall, patients undergoing segmentectomy and wedge resection had no significant different OS and LCSS both in the invasive adenocarcinoma group and the squamous cell carcinoma group. Segmentectomy was associated with improved OS (hazard ratio = 0.626, 95% confidence interval: 0.457-0.858, *P* = 0.004) and LCSS (hazard ratio = 0.643, 95% CI: 0.440-0.939, *P* = 0.022) in invasive adenocarcinoma patients ≤ 65 years old. In patients with ≤ 2 cm invasive adenocarcinoma, segmentectomy was associated with significantly better OS (hazard ratio = 0.811, 95% confidence interval: 0.666-0.988, *P* = 0.038).

**Conclusion:** Survival following segmentectomy or wedge resection was generally equivalent in lung invasive adenocarcinoma and squamous cell carcinoma. However, invasive adenocarcinoma patients who were ≤ 65 years or had tumors ≤ 2 cm in size may have improved survival outcomes after segmentectomy.

## INTRODUCTION

The National Lung Screening Trial (NLST) results encouraged the early detection of lung cancer by computed tomography (CT) screen [1]. In 1995, the Lung Cancer Study Group (LCSG) published the only randomized clinical trial on surgical approaches for stage IA non-small cell lung cancer (NSCLC) [2]. This trial demonstrated that lobectomy was associated with improved survival outcomes compared to limited resection (segmentectomy and wedge resection) [2]. Since then, a number of non-randomized studies, especially studies from Japan, showed equivalent efficacy with limited resection in selected early stage NSCLC [3-7]. A recent study further found that the equivalency of limited resection versus lobectomy varies with tumor histology (ie, invasive adenocarcinoma and squamous cell carcinoma) [8]. However, only a few studies compared the efficacy between the two sublobar resection types, segmentectomy and wedge resection, providing insufficient evidence regarding selection of appropriate limited resection types in early stage lung cancer [5, 6, 9, 10].

In this study, we used the Surveillance, Epidemiology, and End Results (SEER) database to investigate the survival outcomes following segmentectomy versus wedge resection in stage I ≤3 cm lung invasive adenocarcinoma and squamous cell carcinoma.

## RESULTS

A total of 5880 patients with invasive lung adenocarcinoma or squamous cell carcinoma were included in this study, of which 1156 received segmentectomy. Among patients with invasive lung adenocarcinoma, patients undergoing segmentectomy were more likely to be female, had a larger tumor size, had tumors located in the lower lobe, had more lymph nodes examined, and were less likely to receive postoperative radiation therapy. Patients with squamous cell carcinoma receiving segmentectomy were also associated with a larger tumor size, more lymph nodes examined and a lower tendency to receive postoperative radiation therapy. Detailed patient characteristics were listed in Table 1. After adjustment for propensity scores, baseline characteristics were well balanced among patients receiving segmentectomy or wedge resection.

**Table 1:** Characteristics of stage I ≤3 cm lung invasive adenocarcinoma and squamous cell carcinoma patients undergoing segmentectomy versus wedge resection

Variable	Invasive Adenocarcinoma				Squamous Cell Carcinoma			
	Wedge n=3145	Seg n=786	*P*		Wedge n=1579	Seg n=370	*P*	
			Unadjusted	Adjusted			Unadjusted	Adjusted
**Age (years)**			0.841	0.886			0.414	0.641
Mean	69.1	69.0			71.7	71.3		
SD	9.7	9.5			8.3	8.1		
**Gender**			**0.002**	0.528			0.637	0.653
Female	1771 56.3%	490 62.3%			768 48.6%	185 50.0%		
Male	1374 43.7%	296 37.7%			811 51.4%	185 50.0%		
**Race**			0.051	0.333			0.129	0.146
White	2739 87.1%	669 85.1%			1416 89.7%	324 87.6%		
African American	287 9.1%	72 9.2%			116 7.3%	38 10.3%		
Others and unknown	119 3.8%	45 5.7%			47 3.0%	8 2.2%		
**Marital status**			0.132	0.681			0.100	0.901
Married	1668 53.0%	442 56.2%			794 50.3%	206 55.7%		
Unmarried	1348 42.9%	321 40.8%			728 46.1%	156 42.2%		
Unknown	129 4.1%	23 2.9%			57 3.6%	8 2.2%		
**Year of diagnosis**			0.587	0.789			0.415	0.318
1998-2004	1072 34.1%	276 35.1%			566 35.8%	141 38.1%		
2005-2012	2073 65.9%	510 64.9%			1013 64.2%	229 61.9%		
**Tumor size (mm)**			**< 0.001**	0.808			**0.001**	0.801
Mean	17.2	18.6			18.2	19.5		
SD	6.3	6.5			6.4	5.9		
**Lobar distribution**			**< 0.001**	0.237			0.323	0.301
Upper	2057 65.4%	483 61.5%			1045 66.2%	242 65.4%		
Middle	124 3.9%	15 1.9%			51 3.2%	7 1.9%		
Lower	964 30.7%	288 36.6%			483 30.6%	121 32.7%		
**Differentiation**			0.988	0.683			0.432	0.425
Well	606 19.3%	149 19.0%			79 5.0%	11 3.0%		
Moderate	1481 47.1%	378 48.1%			744 47.1%	181 48.9%		
Poor	839 26.7%	204 26.0%			668 42.3%	160 43.2%		
Undifferentiated	22 0.7%	6 0.8%			17 1.1%	5 1.4%		
Unknown	197 6.3%	49 6.2%			71 4.5%	13 3.5%		
**Number of LNs examined**			**< 0.001**	NA			**< 0.001**	NA
≤ 6	2594 82.5%	573 72.9%			1375 87.1%	286 77.3%		
7 or more	367 11.7%	137 17.4%			129 8.2%	63 17.0%		
Unknown	184 5.9%	76 9.7%			75 4.7%	21 5.7%		
**Radiation**			**0.009**	NA			**0.013**	NA
Yes	206 6.6%	32 4.1%			122 7.7%	15 4.1%		
No	2939 93.4%	754 95.9%			1457 92.3%	355 95.9%		

Abbreviations: Seg, segmentectomy; SD, standard deviation; LN, lymph nodes; NA, not adjusted.

In the propensity score-matched analysis, patients undergoing segmentectomy and wedge resection had no significant different OS and LCSS both in the invasive adenocarcinoma group and the squamous cell carcinoma group (Table 2). We further performed subgroup analysis according to age and tumor size. Segmentectomy was associated with improved OS (HR = 0.626, 95% CI: 0.457-0.858, *P* = 0.004) and LCSS (HR = 0.643, 95% CI: 0.440-0.939, *P* = 0.022) in invasive adenocarcinoma patients ≤ 65 years old. In patients with ≤ 2 cm invasive adenocarcinoma, segmentectomy was associated with significantly better OS (HR = 0.811, 95% CI: 0.666-0.988, *P* = 0.038) and a tendency towards better LCSS (HR = 0.813, 95% CI: 0.632-1.047, *P* = 0.109). OS and LCSS were not significantly different between the two resection types in invasive adenocarcinoma patients older than 65 years or had tumors 2-3cm in size. Squamous cell carcinoma patients receiving segmentectomy or wedge resection had comparable OS and LCSS in all the subgroup analysis.

**Table 2:** Propensity score-matched analysis comparing overall survival and lung cancer-specific survival following segmentectomy versus wedge resection

Model	Invasive Adenocarcinoma				Squamous Cell Carcinoma			
	OS		LCSS		OS		LCSS	
	HR 95% CI	*P*	HR 95% CI	*P*	HR 95% CI	*P*	HR 95% CI	*P*
**All patients**	0.871 0.746-1.016	0.078	0.918 0.755-1.116	0.388	1.001 0.818-1.225	0.993	1.082 0.818-1.430	0.582
**Age (yrs)**								
≤ 65	0.626 0.457-0.858	**0.004**	0.643 0.440-0.939	**0.022**	0.715 0.417-1.224	0.221	0.749 0.355-1.583	0.449
> 65	0.997 0.835-1.192	0.978	1.040 0.827-1.309	0.736	0.981 0.786-1.224	0.865	1.042 0.749-1.448	0.808
**Size (cm)**								
≤ 2	0.811 0.666-0.988	**0.038**	0.813 0.632-1.047	0.109	0.915 0.659-1.271	0.597	0.993 0.621-1.585	0.975
2-3	0.865 0.674-1.111	0.256	1.083 0.792-1.482	0.616	0.860 0.633-1.168	0.333	0.738 0.485-1.122	0.156

Abbreviations: OS, overall survival; LCSS, lung cancer-specific survival; HR, hazard ratio; CI, confidence interval.

## DISCUSSION

Although the only published RCT supported the superiority of lobectomy over limited resection in T1N0 NSCLC [2], there is increasing evidence indicating an equivalent efficacy of sublobar resection (segmenetectomy or wedge resection) in selected early-stage patients [3-7]. Compared to segmentectomy, wedge resection is technically a much simpler approach including the resection of lung tumor as well as the surrounding normal lung tissues. However, segmentectomy is still preferred by many surgeons as it is considered to be an anatomical resection with more extensive lymph nodes dissection. Currently, only a few studies compared the survival outcomes following segmentectomy or wedge resection, with relatively smaller samples and conflicting results [5, 6, 9, 10]. Therefore, we undertook this population-based study to investigate the efficacy of these two sublobar resection types after propensity score matching and stratified the analysis in invasive adenocarcinoma and squamous cell carcinoma.

The OS and LCSS were comparable between the two groups when the whole cohort of invasive adenocarcinoma patients were analyzed. Segmentectomy was associated with improved OS and LCSS in invasive adenocarcinoma patients 65 years or younger, but not in the group of patients older than 65 years. Young patients with lung cancer generally have better overall survival as well as lung-cancer specific survival than their older counterparts [14, 15]. Since elder patients usually had a shorter life expectancy along with higher postoperative morbidity and mortality rates, they were considered by some as reasonable candidates for limited resections. Our results further indicated the role of age as a criterion for selection between the two sublobar resection types.

The RCT by LCSG was criticized by some as it included tumors 2-3 cm in size [2]. Some subsequent studies suggested that sublobar resection was a reasonable alternative for stage I lung cancer ≤ 2 cm in size [3-5, 16-18]. Based on these findings, there are two ongoing trials (JCOG0802 [19] and CALGB 140503) comparing sublobectomy to lobectomy in stage I ≤ 2 cm lung cancer. We found that in ≤ 2 cm invasive adenocarcinoma, OS following segmentectomy was significantly better than that after wedge resection, while survival outcomes were comparable between patients receiving segmentectomy or wedge resection in invasive adenocarcinoma 2-3 cm in size, supporting the application of segmentectomy in small-sized invasive lung adenocarcinoma.

Compared to lung adenocarcinoma, squamous cell carcinomas are more likely to show local aggressiveness and invasion of adjacent structures. To the best of knowledge, our study is the first to compare segmentectomy to wedge resection in this lung cancer histologic subtype. We found comparable OS and LCSS following segmentectomy or wedge resection in patients with squamous cell carcinoma in the whole cohort as well as in subgroup analysis stratified by age and tumor size, suggesting an equivalent efficacy of the two limited resection types in these tumors.

In conclusion, survival following segmentectomy or wedge resection was generally equivalent in lung invasive adenocarcinoma and squamous cell carcinoma. However, invasive adenocarcinoma patients who were ≤ 65 years or had tumors ≤ 2 cm in size may have improved survival outcomes after segmentectomy.

## MATERIALS AND METHODS

We used the SEER*Stat software version 8.2.1-alpha to obtain the SEER database (1988-2012) through on-line access. This study was approved by the Institutional Review Board of Fudan University Shanghai Cancer Center.

We only included patients with lung cancer as the first primary malignancy. As SEER codes for wedge resection (Code 21) or segmentectomy (Code 22) were not available until 1998, patients diagnosed before 1998 were excluded. Tumors should be stage I and ≤ 30 mm in diameter. Cases were excluded if they did not have sufficient information on tumor size or pathologic stage, or if they received radiation therapy prior to surgery. In the current lung adenocarcinoma classification proposed by the International Association for the Study of Lung Cancer, the American Thoracic Society, and the European Respiratory Society (IASLC/ATS/ERS) [11], non-mucinous bronchioloalveolar carcinoma (BAC) could potential be adenocarcinoma in situ (AIS) or minimally invasive adenocarcinoma (MIA). As AIS and MIA have extremely good survival rates after resection [12, 13], we excluded non-mucinous BAC and only included invasive adenocarcinoma and squamous cell carcinoma in this study. Patients with other NSCLC histologic types were excluded because of limited number of cases. The following clinicopathologic data were retrieved in this study: age at diagnosis, gender, ethnicity, marital status, year of diagnosis, tumor location, type of surgical resection, number of lymph nodes examined, tumor size, disease stage, tumor histology, tumor grade, information of radiation therapy, overall survival (OS) and lung cancer-specific survival (LCSS).

### Statistical analysis

We used Pearson's chi-squared test or Fisher's exact test to assess the association between type of resection and a categorical variable. Age and tumor size were treated as continuous variables and were compared using independent sample *t* test. To adjust for the potential differences in the baseline characteristics between patients undergoing wedge resection or segmentectomy, we used propensity score matching methods. Propensity scores were calculated using logistic regression including preoperative variables: age, sex, race, year of diagnosis, marital status, tumor location, size and grade. Patients receiving segmentectomy and wedge resection were matched 1:1 based on their propensity scores, and Cox regression multivariate survival analysis adjusting for all the clinicopathologic variables was performed to compare the OS and LCSS of patients receiving segmentectomy versus wedge resection. Hazard ratio (HR) and its 95% confidence interval (CI) along with *P* values were calculated. All the tests were conducted in Stata (version SE/11, StataCorp, Texas). A two-tailed *P* value < 0.05 was set as statistically significant.
